# Editorial

**DOI:** 10.1515/iss-2022-2001

**Published:** 2022-07-18

**Authors:** Udo Rolle

**Affiliations:** Department of Pediatric Surgery and Pediatric Urology, University Hospital Frankfurt/M., Frankfurt, Germany

Pediatric surgery is an independent specialty providing surgical care to patients from newborn age until young adulthood. One of the main areas in this discipline deals with the correction of congenital anomalies. Other important areas of pediatric surgery are pediatric gastrointestinal and hepatobiliary surgery, pediatric urology, pediatric oncological surgery and pediatric orthopedic surgery. The scientific articles presented within this special issue of *Innovative Surgical Sciences* include some excellent examples of the comprehensive medical care as well as the academic spectrum of pediatric surgery.

The paper by Derikx et al. contains a well-conducted systematic review and meta-analysis on the incidence of various forms of ileus following surgery for congenital abdominal malformations. This article shows that ileus is a common complication following surgery of congenital anomalies of the abdomen, requiring immediate treatment. Further research is warranted to identify risk factors and potential preventative measures.

The paper by Uecker et al. deals with an indispensable aspect in the management of patients with congenital malformations, which is the transition of care. Nowadays, survival is no longer a major outcome measure in this cohort of patients, rather long-term quality of life is more significant. The provided overview summarizes relevant surgical-treated congenital anomalies, focusing on long-term morbidity and quality of life assessment to demonstrate the necessity of a structured and standardized transition of care.

Another major aspect of pediatric surgery, namely innovation in surgical techniques, are presented by Privitera at al. Their paper describes novel approaches for intraoperative visualization and image-guided surgery. The utilization of these newly developed technologies in pediatric surgery will lead to a better and more detailed visualization of small anatomical structures, resulting in less surgical complications and improved outcome.

I wish to thank the journal to for giving the opportunity to showcase important aspects of clinical care and surgical innovations in pediatric surgery.

